# Preconception parental personality disorder and psychosocial outcomes during the perinatal period: a prospective population-based study

**DOI:** 10.1007/s00127-025-02968-3

**Published:** 2025-07-29

**Authors:** Claire A. Wilson, Hanafi Mohamad Husin, S. Ghazaleh Dashti, Raquel Catalao, Rohan Borschmann, Stephanie Brown, Louise M. Howard, Jessica A. Kerr, Jonathan Monk-Cunliffe, Paul Moran, George C. Patton, Craig A. Olsson, Elizabeth Spry

**Affiliations:** 1 Institute of Psychiatry, Psychology and Neuroscience, King’s College London and South London and Maudsley NHS Foundation Trust, London, UK; 2 Centre for Adolescent Health, Murdoch Children’s Research Institute, Melbourne, Australia; 3 Clinical Epidemiology and Biostatistics Unit and Centre for Adolescent Health, Murdoch Children’s Research Institute and Royal Children’s Hospital, Melbourne, Australia; 4 South West London and St George’s Mental Health NHS Trust and Section of Women’s Mental Health, Institute of Psychiatry, Psychology and Neuroscience, King’s College London, London, UK; 5 Murdoch Children’s Research Institute, Royal Children’s Hospital and University of Melbourne, Melbourne, Australia; 6 School of Population Health, Curtin University, Perth, Australia; 7 Department of Psychiatry, University of Oxford and Oxford Health NHS Foundation Trust, London, UK; 8 Section of Women’s Mental Health, Institute of Psychiatry, Psychology and Neuroscience, King’s College London, London, UK; 9 Department of Paediatrics, University of Melbourne, Melbourne, Australia; 10 Department of Psychological Medicine, University of Otago Christchurch, Melbourne, Australia; 11 Centre for Academic Mental Health, Population Health Science Institute, University of Bristol, Bristol, UK; 12 Deakin University, Centre for Social and Early Emotional Development, School of Psychology, Geelong, Australia

**Keywords:** Preconception, Perinatal, Personality disorder, Mental health, Social support

## Abstract

**Purpose:**

Birthing individuals with personality disorder in young adulthood before pregnancy may be at increased risk of potentially modifiable adverse outcomes in the perinatal period that impact parental and child health. We aimed to investigate the perinatal psychosocial outcomes of preconception (prior to pregnancy) personality disorder.

**Methods:**

Prospective analysis of 398 birthing individuals with 609 infants from Victorian Intergenerational Health Cohort Study (VIHCS). Preconception personality disorder was measured using the Standardised Assessment of Personality (SAP) at age 24. A range of parental outcomes were assessed during pregnancy and at one year postpartum (age 28 to 37). Log-binomial generalised estimating equations were used to estimate univariable associations between preconception personality disorder and each perinatal outcome.

**Results:**

Individuals with preconception personality disorder (compared to those without) were approximately two times more likely to have antenatal anxiety symptoms (risk ratio (RR) 2.08, 95% confidence interval (CI) 1.19–3.65) and reduced social support (antenatal RR 2.01, 95% CI 0.98–4.13; postnatal RR 1.38, 95% CI 0.91–2.10). Weaker associations were also observed for experiencing stressful life events (RR 1.37, 95% CI 0.98–1.90) and, albeit with less certainty, for poorer partner relationship quality (RR 1.44, 95% CI 0.78–2.64) and depressive symptoms (antenatal RR 1.56, 95% CI 0.84–2.91; postnatal RR 1.44, 95% CI 0.73–2.83). Close to null associations were observed for parents’ self-efficacy or perceived parent-infant bond.

**Conclusion:**

The findings highlight a group who may be vulnerable to multiple adverse perinatal outcomes; those with personality disorder and their families may benefit from additional support both with pregnancy planning and into parenthood.

**Supplementary Information:**

The online version contains supplementary material available at 10.1007/s00127-025-02968-3.

## Introduction

Personality disorder is characterised by problems in functioning relating to aspects of the self (such as identity and self-worth) and/or interpersonal dysfunction that have persisted over an extended period of time [1]. Personality disorder has an estimated global prevalence of between 6% and 8% [2, 3]. It is a condition that is often co-morbid with other mental disorders and is associated with premature mortality and functional difficulties across the life course [2]. There is substantial debate around the definition and diagnostic criteria for personality disorder, with some asserting that personality dysfunction may be more usefully conceptualised as dimensional, on a continuum from ‘normal’ personality to severe personality disorder. Some individuals may exhibit ‘traits’ of the disorder without meeting all the diagnostic criteria. Indeed, individuals who exhibit such ‘traits’ but who do not meet criteria for personality disorder are now formally recognised as having ‘personality difficulty’ in the International Classification of Diseases 11th edition (ICD-11) [1].

Young adulthood is a time of peak fertility and in many parts of the world is associated with transition to parenthood. We have previously reported that as many as one in five community-dwelling young adults (mean age 24 years) were affected by personality disorder when systematically assessed using a diagnostic interview [4].

Studies of those in the perinatal period (pregnant or up to one year postpartum), in which personality disorder is systematically measured, have found prevalence of personality disorder to be between 4.9% and 12.6% [5] and of elevated personality disorder traits 16.2% [6]. There is some prior evidence that those with personality disorder or personality disorder traits experience adverse outcomes during the perinatal period. There are a range of vulnerabilities that increase the risk for adverse outcomes, for example unplanned pregnancy [7]. In a South London community-based sample, elevated levels of personality disorder traits were associated with insecure accommodation status and thoughts of self-harm during pregnancy [6]. Moreover, during the perinatal period, those with elevated personality disorder traits and personality disorder are more likely to develop the common mental disorders of depression and anxiety [6, 8].

Theoretical frameworks of personality disorder often focus on the interpersonal difficulties and the associated difficulties with reciprocity [9]. Elevated personality disorder traits have been found to predict lower parental* sensitivity and difficulties with parent-infant interaction [10]. Such difficulties may have their origins in the childhood trauma which is common in the aetiology of personality disorder [11]. There may also be difficulties within the wider social environment, including a range of stressors such as exposure to violence and abuse and socio-relational difficulties, during a time of life in which social support is particularly important.

However, most of the research to date on the perinatal outcomes of those with personality disorder, particularly those related to parenting, have focussed exclusively on borderline personality disorder [11]. Additionally, prior studies typically use clinical or high-risk samples, not reflecting needs across the broader community. Finally, given that personality disorder is not stable (with only 50% of cases enduring [12]), there is a case for identifying personality disorder before pregnancy, providing an opportunity for earlier intervention during pregnancy planning and support in young adulthood prior to conception.

Therefore there is a need to better understand the perinatal outcomes of those with the full range of personality disorders and personality disorder traits, identified well before pregnancy, in a population-based community sample. This knowledge could illuminate potential foci for intervention to mitigate the impact of these early adversities on birthing individuals with personality disorder and their children. The aim of this study was to describe the psychosocial outcomes in pregnancy and the postpartum for parents with preconception (prior to pregnancy) personality disorder or elevated personality disorder traits in a community sample of individuals assessed before, during and after pregnancy.

*the words ‘parent’ and ‘parental’ refer specifically to birthing parents throughout.

## Methods

### Sample

The Victorian Intergenerational Health Cohort Study (VIHCS) is a prospective study (beginning in 2006) of preconception predictors of infant and child health, described elsewhere [13]. It arose from the Victorian Adolescent Health Cohort Study (VAHCS), which comprises 1943 mid-secondary school students from the state of Victoria, Australia (1000 were female sex at birth) [14]. VAHCS participants were selected via a two-stage cluster sampling design and assessed six-monthly during adolescence (VAHCS waves one to six: mean ages 14·9 to 17·4 years) and three times during young adulthood (VAHCS waves seven to nine: 20·7, 24·1 and 29·1 years of age). Beginning during the ninth wave of VAHCS, participants were screened every six months for pregnancies via SMS, email and phone (between 2006 and 2013) During this time, participants were 29 to 35 years of age, which included median maternal ages for Australian births at the time (Australian Bureau of Statistics, 2013). Participants reporting a pregnancy or recently born infant were interviewed during trimester three and/or at two months and one year postpartum for every infant born during the VIHCS screening period.

### Measures

#### Preconception exposure (VAHCS)

The Standardised Assessment of Personality (SAP) was administered during VAHCS wave eight. The SAP is a diagnostic informant interview that assesses the presence of ten categories of DSM-IV personality disorder [15]. It has high inter-rater (kappa 0.76) and good temporal (kappa 0.65) reliability [16]. Participants nominated a friend, partner or family member to complete the SAP via a structured computer assisted telephone interview with a trained research assistant; informant- (as opposed to self-) report removes the impact of current mental state on the description of personality [17]. Interviewers asked the informant to describe the participant using a series of prompts and probing questions, recording relevant adjectives for specific categories of personality disorders (for example ‘manipulative’ for narcissistic personality disorder). If one or more adjectives were identified, further questions for that category were asked. Each question to which the informant responded ‘yes’ was recorded as one trait score. If three or more trait scores were recorded for each personality disorder, the functional impact was also assessed across three domains: personal distress, occupational impairment and social impairment [15].

Responses to the SAP were utilised as follows:

Any personality disorder (binary): defined as having at least one disorder with trait scores at or above threshold and the presence of at least one impairment (personal distress, occupational impairment or social impairment) [15].

Personality disorder traits: total trait score (out of 80). This was generated by summing scores across all ten categories of personality disorder then standardising to allow effect sizes to be interpreted in unit increase of standard deviation.

Two additional binary exposure derivations were used as secondary exposures to examine the associations by type and severity of personality disorder:

Cluster: personality disorders were grouped according to DSM-IV clusters A (paranoid, schizoid, and schizotypal), B (antisocial, borderline, histrionic, and narcissistic) and C (avoidant, dependent, and obsessive-compulsive) [18].

Severity: following the Tyrer and Johnson method [19], consisting of four categories- (1) no personality disturbance: trait scores of at least two lower than the threshold across all categories of personality disorder, (2) personality difficulty: trait score of only one lower than threshold for any personality disorder(s), (3) simple personality disorder: trait score at or above threshold for personality disorder(s) in one DSM-IV cluster only and (4) complex personality disorder: trait scores at or above threshold for personality disorders in two or three DSM clusters.

#### Perinatal outcomes (VIHCS)

Outcomes were assessed in trimester three of pregnancy (VIHCS wave one) and/or at one year postpartum (VIHCS wave three) via participant self-report, using structured computer-assisted telephone interviews. These were expressed as binary outcomes for all primary analyses across the three domains of: (1) perinatal mental health, consisting of depressive symptoms (elevated symptoms defined as ≥ 10: yes versus no) and thoughts of self-harm (any versus none) on the Edinburgh Postnatal Depression Scale [20] and anxiety symptoms (elevated symptoms defined as ≥ 2: yes versus no) on the Clinical Interview Schedule (CIS) Anxiety Subscale [21, 22]; (2) factors that may lead to or contribute to poorer perinatal psychosocial outcomes, i.e. any stressful life events defined as score ≥ 1, versus none, on the List of Threatening Experiences [23], low-medium social support defined as score ≤ 24, versus high social support, on the Maternity Social Support Scale [24, 25] and low-medium partner relationship quality defined as score ≤ 3, versus high relationship quality, on the Dyadic Adjustment Scale [26]; (3) perceptions of parenting, comprising the felt parent-infant bond (bonding difficulties defined as score ≥ 2: yes versus no) on the Postpartum Bonding Questionnaire [27] and parental self-efficacy (low-medium versus high) [28]. Secondary analyses utilised continuous scores standardised to facilitate comparison of effect sizes between outcomes. Further information on these measures and how they were dichotomised are provided in Supplementary Table 1.

### Statistical analysis

Analyses were performed in Stata version 18 [29]. Descriptive statistics were summarised as mean and standard deviation (SD) for continuous variables and frequency and percent for binary/categorical variables.

To account for clustering by family i.e. individuals with more than one pregnancy in the cohort, log-binomial generalised estimating equations (GEE) with an exchangeable correlation matrix were used to estimate the risk ratios (RRs) and 95% confidence intervals (CIs) for the association between each personality disorder exposure and outcome. Additional analyses utilising standardised continuous outcome scores were conducted using linear GEE to produce standardised beta coefficients with 95% CIs. All analyses were univariable, consistent with the study’s aim to describe perinatal outcomes for individuals with personality disorder [30].

The proportion of missingness within each variable is displayed in Table. For the primary analysis, missing data were handled using multiple imputation by chained equations [31] and generating 50 imputed datasets (50% of participants had missing data on one or more variables [32]). All analysis variables were included in the imputation models, in addition to the following auxiliary variables: mother born outside Australia, grandparent education, grandparent smoking and socioeconomic status (Australian Bureau of Statistics - Socio-economic Indexes for Areas 2006; SEIFA 2006), all of which were predictive of non-response. Final estimates and 95% CIs were obtained by pooling results using Rubin’s rules [33]. Available case analyses were also conducted as secondary analyses.

## Results

**Fig. 1:**
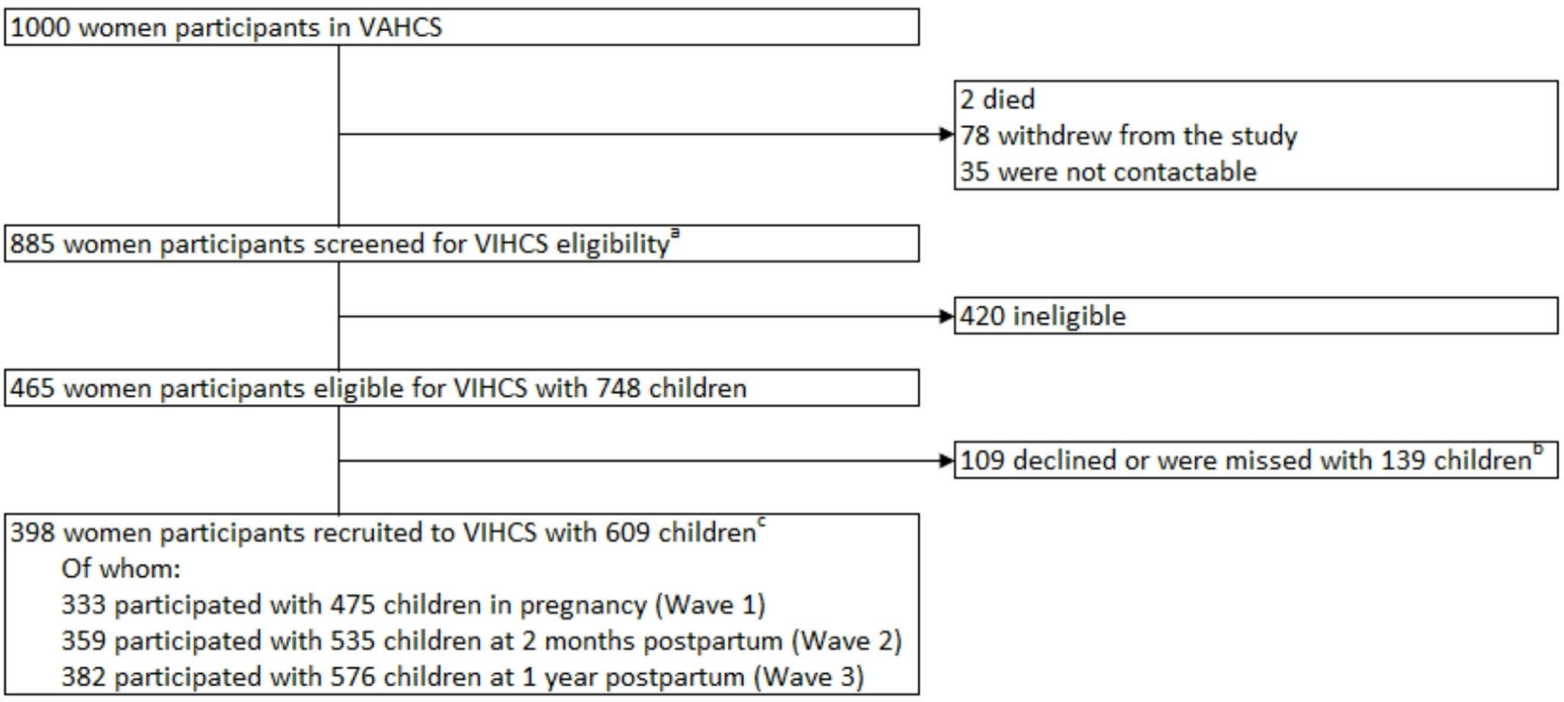
Sampling of the Victorian Intergenerational Health Cohort Study (VIHCS) from 2006–2014. VAHCS– The Victorian Adolescent Health Cohort; VIHCS– The Victorian Intergenerational Health Cohort. ^a^Eligibility for study entry at VIHCS wave 1 defined as a viable pregnancy in trimester three during VIHCS screening (1 September 2006 to 30 June 2013); eligibility at VIHCS wave 2/3 defined as a live birth during VIHCS screening. ^b^Of the 109 VAHCS participants who didn’t participate for one or more eligible VIHCS children, 67 were completely excluded and the remaining 42 were recruited to participate in the study with at least one other child. ^c^The cohort remained open during Waves 1–3, so that parents could enter the study at Wave 2 or 3 if they had missed earlier wave/s. Many parents participated with more than one child born during the VIHCS recruitment phase

The sample comprised 398 birthing individuals with 609 offspring. The flow of participants through VIHCS is presented in Fig. 1. Demographics of the sample are presented in Table 1. Within this sample, 17% of participants with available data on the SAP met the threshold for personality disorder.

**Fig. 2:**
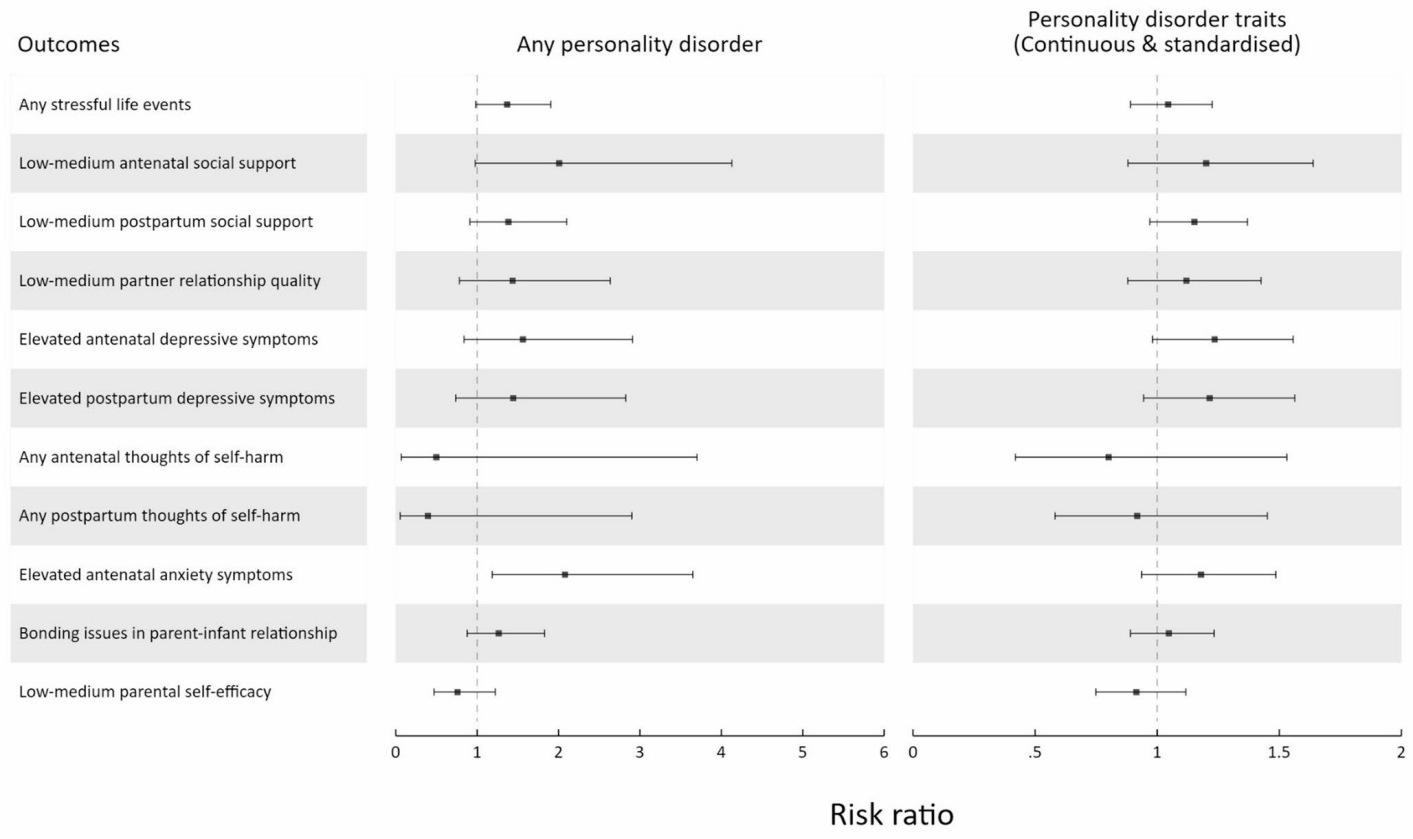
Forest plot for estimated risk ratios with 95% confidence intervals of any personality disorder (compared to no personality disorder) and total personality disorder traits on antenatal and postpartum outcomes. Estimates were obtained from multiply imputed datasets

Figure 2 displays the results from multiply imputed data of the primary analyses utilising the two exposures: presence of any personality disorder and total personality disorder traits. Individuals with preconception personality disorder were approximately two times more likely to have antenatal anxiety symptoms (risk ratio (RR) 2.08; 95% confidence interval (CI) 1.19–3.65) and reduced social support (antenatal RR 2.01; 95% CI 0.98–4.13 and postnatal RR 1.38; 95% CI 0.91–2.10). Weaker associations were also observed for experiencing stressful life events (RR 1.37; 95% CI 0.98–1.90), poorer partner relationship quality (RR 1.44; 95% CI 0.78–2.64) and, albeit with less certainty, for depressive symptoms (antenatal RR 1.56; 95% CI 0.84–2.91 and postnatal RR 1.44; 95% CI 0.73–2.83). Close to null associations were observed for parents’ self-efficacy (RR 0.76; 95% CI 0.47–1.22) and perceived parent-infant bond (RR 1.27; 95% CI 0.88–1.83). For thoughts of self-harm, confidence intervals were wide due to the small number of participants reporting this outcome (antenatal RR 0.50; 95% CI 0.07–3.70 and postnatal RR 0.40; 95% CI 0.05–2.90).

**Fig. 3:**
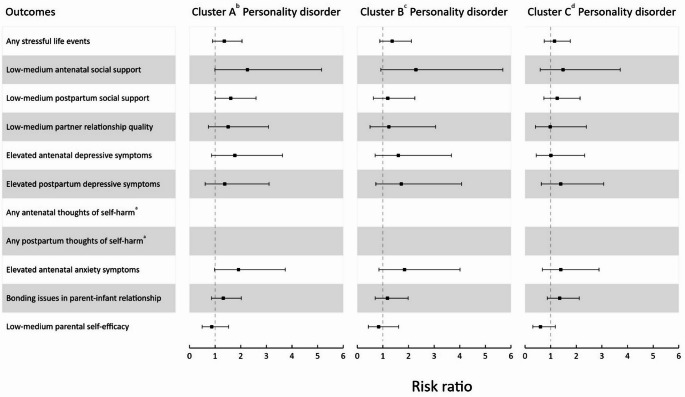
Forest plot for estimated risk ratios with 95% confidence intervals of any personality disorder by DSM cluster (compared to no personality disorder) on antenatal and postpartum outcomes. Estimates were obtained from multiply imputed datasets. ^a^Risk ratios for antenatal and postpartum thoughts of self-harm were unable to be estimated due to non-convergence caused by small cell sizes. ^b^Cluster A (paranoid, schizoid and schizotypal), ^c^Cluster B (antisocial, borderline, histrionic and narcissistic) and ^d^Cluster C (avoidant, dependent and obsessive-compulsive)

**Fig. 4:**
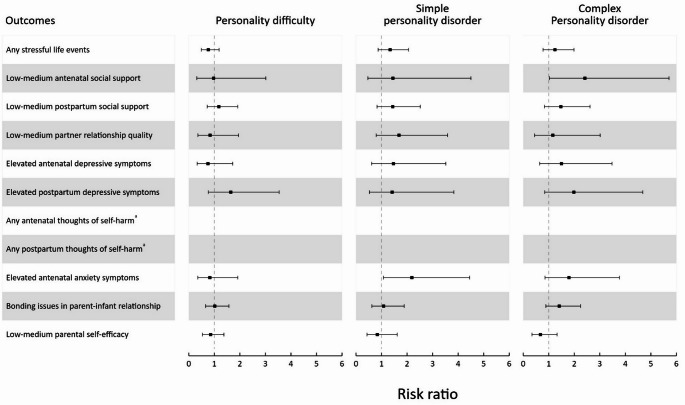
Forest plot for estimated risk ratios with 95% confidence intervals of personality disorder severity (compared to no personality disorder) on antenatal and one-year postpartum outcomes. Estimates were obtained from multiply imputed datasets. ^a^Risk ratios for antenatal and postpartum thoughts of self-harm were unable to be estimated due to non-convergence caused by small cell sizes

Available case analyses yielded a similar pattern of results (Supplementary Tables 2a and 2b). The findings were broadly similar across personality disorder clusters, with little suggestion of a difference between clusters (Fig. 3). However, when examining the influence of personality disorder severity (Fig. 4), larger RRs for adverse outcomes were observed in those with simple or complex personality disorder compared to personality difficulty. Available case analyses yielded a similar pattern of results (Supplementary Tables 3a-c and 4a-c). Supplementary Tables 5a-b, 6a-c and 7a-c display the results of available case analyses utilising standardised continuous scores on the outcomes, which also produced a similar pattern of results.

## Discussion

### Main findings

Using data from 398 birthing individuals within VIHCS, individuals with preconception personality disorder were about two times more likely to have antenatal anxiety symptoms and reduced social support. The associations were weaker for depression, stressful life events and relationship quality and close to null for the parenting outcomes of perceived difficulties with bonding and lower self-efficacy. Larger RRs were observed when considering personality disorder compared to personality difficulty and when considering antenatal outcomes compared with postnatal outcomes.

### Findings in context

The proportion of the sample in this study experiencing personality disorder (17%) has been previously reported in this population [4] and is within range of previously reported estimates [34]. It is consistent with the observation that symptoms of personality disorder may reduce with increasing age [4] but other sources of heterogeneity in prevalence estimates include variation in the diagnostic interview used and its method of administration [3].

The broad range of psychosocial outcomes examined in this analysis is a novel addition to the literature, making it the first study to highlight the somewhat poorer perinatal social outcomes of preconception personality disorder. Theoretical frameworks of personality disorder often focus on interpersonal difficulties [9] and the detected associations with impaired social support are consistent with this. Indeed the negative attitudes towards those with personality disorder often impact social relationships and relationships with healthcare providers. Although this study did not examine relationships with perinatal care providers, a previous study of those with borderline personality disorder reported low levels of engagement with antenatal care [35]. It is interesting that social outcomes did not differ substantially between personality disorder clusters as differences in interpersonal functioning have been observed between the different clusters [36]; it is likely that the more global outcomes assessed in this study did not capture any subtler differences.

Previous studies of the perinatal outcomes of those with personality disorder have predominantly focussed on mental disorders [5, 6, 8], as opposed to the broader set of outcomes examined in this study. As in our study, associations have been observed between personality disorder and symptoms of antenatal anxiety and depression [8] and also between elevated disordered personality traits and a range of mental disorders on diagnostic clinical interview during pregnancy [6].

The other area of focus in the wider literature has been on parenting and the relationship with the infant, particularly among those with borderline personality disorder [11]. In our study, there was limited evidence of an association between personality disorder and perceived parental self-efficacy or the parent’s felt bond with their infant. However, a previous study found that those with disordered personality traits were rated as being less sensitive to their infants’ needs during observed interactions [10]. Our findings suggest that individuals with preconception personality disorder may feel bonded to their infants and capable as parents but in light of prior literature, there may nonetheless be a need for parenting supports in attending to infant socio-emotional needs.

Overall, we found stronger associations of personality disorder and disordered traits with antenatal than postnatal outcomes. There are a range of factors that may have protected against these adverse impacts in the postpartum, such as interventions from health and social care services [37]. Indeed the more limited evidence for adverse postnatal than antenatal outcomes may be as a result of our sample being buffered by interventions focussed more on providing support during the postpartum than during pregnancy [38].

### Strengths and limitations

In addition to examining a wide range of psychosocial outcomes, the comprehensive prospective measure of personality disorder facilitated the investigation of different personality disorder exposures measured prior to pregnancy. As with all cohort studies, the possibility of differential recruitment, attrition and non-response, and changing population demographics over time may have led to under-representation of some population groups. VAHCS has a high retention rate, with 85% of those with live births during screening participating in VIHCS. In the study, missing data were low at preconception and postpartum waves but higher antenatally due to challenges of detecting all pregnancies before birth. In our primary analysis, we handled missing data using multiple imputation and included a range of auxiliary variables that may have predicted non-response in the imputation models to reduce potential bias due to missing data [39].

Some of the exposure groups in the sample, for example personality disorder clusters, had relatively small numbers of participants, reducing precision of our estimates; findings need replication in larger or pooled samples with similarly strong longitudinal designs. It is also important to note limitations to generalisability including the restricted age range of participants (29 to 35 years old) and the relative lack of racial diversity within the sample (Table 1), albeit this represents the baseline population at the time. There is evidence that younger individuals with personality disorder may experience more adverse perinatal outcomes [5]. It is also important to note other sources of diversity of experience in those with personality disorder which may impact perinatal outcomes and which were unmeasured in the study, including previous experiences of pregnancy, parenting and pregnancy loss; less than half of the sample were primiparous (Table 1) [7].

Moreover, the proposed framework for descriptive epidemiology [30] was followed to describe the perinatal psychosocial outcomes for parents with versus without personality disorder. In this context, adjustment for other variables may have led to misleading findings. For example, adjusting for socioeconomic status would have led to estimating RRs for outcomes in a hypothetical world where the two groups of those with versus without personality disorder had a similar socioeconomic position, which is not what we were interested in estimating.

### Clinical and research implications

The findings shed light on the challenging social circumstances that may surround the pregnancies of those with personality disorder and of which maternity professionals should be aware when providing holistic support to this population during the perinatal period. These challenges would be usefully explored further in larger and diverse samples and in discussion with those with personality disorder using qualitative methodology. Many of the psychosocial outcomes considered in this analysis, for example social support, are known protective factors for mental ill health [40]. Moreover, there is evidence that parental personality disorder is associated with adverse neonatal outcomes, including low birth weight and prematurity [41]. Therefore our results provide new insights into possible mediating influences on these adverse outcomes that may arise during (or before) pregnancy and that could be the focus of future interventions to reduce the occurrence of these outcomes. It is important that such interventions are developed alongside those with lived experience of personality disorder and that they are trauma-informed, given the high incidence of trauma among those with personality disorder [11] and that preventative interventions span educational, community, healthcare and social care settings. Future research could also examine these mediating factors as intermediary pathways in the intergenerational transmission of risk, including that for offspring mental ill health [42].

## Conclusions

Our findings advance understanding of the broader set of psychosocial outcomes associated with maternal personality disorder, within a socio-ecological context. They highlight the importance of mental health within preconception interventions [43, 44] and they also underscore the value in shifting the focus solely from parental mental health and parenting interventions to those interventions that also address social and structural factors known to influence parental and child health.

**Table 1:** Descriptive statistics of demographics, exposures and outcomes for N=609 offspring from 398 participants

Demographics	N	n/Mean	%^a^/SD	Missing (%^b^)
Primiparous offspring	609	282	46%	0 (0)
Maternal age at offspring birth	609	32.26	1.80	0 (0)
Australian-born mother	398	340	89%	15 (4%)
Parents of participant did not complete high school	398	152	38%	5 (1%)
SEIFA IRSAD^	398			20 (5%)
1^st^ quintile (most disadvantaged)		32	8.5%	
2^nd^ quintile		35	9.3%	
3^rd^ quintile		65	17.2%	
4^th^ quintile		138	36.5%	
5^th^ quintile (most advantaged)		108	28.6%	

Exposure		n/Mean	%^a^/SD	Missing (%^b^)
Any personality disorder	398	52	17%	92 (23%)
Personality disorder cluster	398			
Any Cluster A personality disorder		28	9%	
Any Cluster B personality disorder		20	7%	
Any Cluster C personality disorder		28	9%	
Personality disorder severity	398			
No personality disturbance		184	60%	
Personality difficulty		70	23%	
Simple personality disorder		31	10%	
Complex personality disorder		21	7%	
Total personality disorder symptoms	398	8.56	9.35	

Outcome		n	%^a^	Missing (%^b^)
Stressful life events (antenatal)	609	132	32%	195 (32%)
Social Support (antenatal)	609	29	7%	192 (32%)
Social Support (postpartum)	609	118	20%	33 (5%)
Partner relationship quality (antenatal)	609	44	11%	200 (33%)
Depressive symptoms (antenatal)	609	47	11%	191 (31%)
Depressive symptoms (postpartum)	609	46	8%	34 (6%)
Thoughts of self-harm (antenatal)	609	9	2%	191 (31%)
Thoughts of self-harm (postpartum)	609	14	2%	34 (6%)
Anxiety symptoms (antenatal)	609	54	13%	191 (31%)
Parent-infant felt bond (postpartum)	609	149	26%	33 (5%)
Parental self-efficacy (postpartum)	609	119	21%	35 (6%)

^SEIFA IRSAD: Socio Economic Index for Areas - Index of Relative Socioeconomic Advantage and Disadvantage

^a^Percentage based on available data

^b^Percentage based on total sample

## Electronic supplementary material

Below is the link to the electronic supplementary material.

Supplementary Material 1

## Data Availability

The data that support the findings of this study are available on reasonable request from the senior authors. The data are not publicly available as they contain information that could compromise research participant privacy/consent.
