# Supplementary material for: Preconception parental personality disorder and psychosocial outcomes during the perinatal period: a prospective population-based study
Source: Soc Psychiatry Psychiatr Epidemiol. 2025 Jul 29;61(2):391–401. doi: 10.1007/s00127-025-02968-3 (PMC12948797; doi:10.1007/s00127-025-02968-3)
Supplement: Supplementary file 1 — Supplementary Material 1 [file 127_2025_2968_MOESM1_ESM.docx]

*Supplementary Table 1: Description of antenatal and one year postpartum outcomes*

| **Outcome** | **Description** | **Time assessed** |
| --- | --- | --- |
| Stressful life events | The List of Threatening Experiences [1] consists of 12 categories of events such as illness, death, relationship difficulties, financial hardship and encounters with crime that may affect the participant or those close to them in the past 12 months. Participants who responded ‘yes’ further reported the level of distress the event had caused them (responses from 0 (none) to 3 (very much)) and how long the distress lasted in weeks. A total score was created by summing the number of life events where participants reported experiencing distress levels at ‘quite a lot’ or ‘very much’. This was dichotomised into any stressful life events (≥1) with a reference group of no stressful life events (0). The measure has been found to have good 6- and 3- month test-retest reliability (κ = 0.96 and 0.88 respectively) and concurrent validity (6=month sensitivity = 0.89, specificity = 0.74; 3-month sensitivity = 1.0, specificity = 0.88) [1]. | Trimester 3 |
| Social support | The Maternity Social Support Scale [2,3] consists of 6 items about how often the participant feels supported and loved by family, friends and partner and how often there is conflict with the partner. Item responses ranged from 1 (never) to 5 (always). Items 4 and 5 were reverse scored and participants who were not in a relationship were not asked items 3-6 about partner social support. A total score was calculated from the responses across all applicable items, where higher scores indicate greater social support. The variable was dichotomised into low to medium social support (scores of 0-24) with a reference group of high social support (scores of 25-30). The Maternity Social Support Scale has demonstrated good predictive validity [2]. | Trimester 3 & 1 year postpartum |
| Partner relationship quality | The Dyadic Adjustment Scale [4] consists of 7 items about intimate relationship satisfaction measuring dyadic satisfaction, dyadic consensus, dyadic cohesion and affectional expression. Item responses ranged from 0 (all the time) to 5 (never). Items 1 and 6 were reverse coded. A mean score was calculated from the responses across all items, where higher scores indicate greater relationship satisfaction. This was dichotomised into low to medium relationship satisfaction (scores of <4) with a reference group of high relationship satisfaction (scores of ≥4). The Dyadic Adjustment Scale has demonstrated high internal consistency (α = 0.96) and construct validity when assessed against other widely-used measures of partner relationship quality (*r* = 0.86) [4]. | Trimester 3 |
| Depressive symptoms | The Edinburgh Postnatal Depression Scale (EPDS) [5] consists of 10 items about symptoms of depression. Item responses ranged from 0 to 3. Items 1, 2 and 4 were reverse scored and an overall total score was calculated by summing scores for all items, where higher scores indicate more depressive symptoms. A cut-off value of 10 was used to indicate elevated depressive symptoms [5,6] with a reference group of scores <10. The EPDS has demonstrated good internal consistency (α = 0.87), and this threshold has been recommended for use in detecting mild to severe postnatal depression, with specificity ranging from 0.78-0.84 and sensitivity ranging from 0.84-0.95 against semi-structured and structured diagnostic interviews [5]. | Trimester 3 & 1 year postpartum |
| Thoughts of self-harm | Thoughts of self-harm were assessed using a single item from the EPDS (as above): ‘The thought of harming myself has occurred to me’. Item response ranged from 0 (never) to 3 (quite often). This was expressed as any thoughts of self-harm (≥1) with a reference group of no thoughts of self-harm (0). | Trimester 3 & 1 year postpartum |
| Anxiety symptoms | The Clinical Interview Schedule (CIS) [7,8] Anxiety Subscale consists of 2 screening items and 8 items to assess anxiety symptoms. A total score was calculated by summing the responses to the 8 items, ranging from 0 to 8. Higher scores indicate more anxiety symptoms. The variable was dichotomised into elevated anxiety symptoms (scores of 2-8) with a reference group of no anxiety symptoms (<1). It has adequate reliability ( 0.74-0.90) and validity against psychiatrist-rated scores using structured interviews and clinical judgment (*r* = 0.70) [7]. | Trimester 3 |
| Parent-infant felt bond | The Postpartum Bonding Questionnaire (PBQ) [9] consists of 25 items measuring four domains: general impaired bonding, rejection and anger, infant-focused anxiety and risk of abuse. Item responses ranged from 0 (always) to 5 (never). All items were reverse scored such that higher scores indicated greater parent-infant bonding and a total score was calculated for each bonding factor. This was dichotomised into ‘bonding issues’ (≥2 problem areas of bonding) with a reference group of ‘no bonding issues’ (0-1 problem areas of bonding). The  scale has demonstrated adequate internal consistency (α = 0.63–0.79) and test-retest reliability (0.77-0.95), and predicts diagnosed bonding disorders with sensitivity 0.84 and specificity 0.74 [9,10]. | 1 year postpartum |
| Parental self-efficacy | 4 items were adapted from the Longitudinal Study of Australian Children (LSAC) [11]. These items enquire about confidence in ability as a parent. Item responses ranged from 1 to 5, where higher scores indicated greater self-efficacy. A mean score was calculated then dichotomised into low-medium parental self-efficacy (scores of <4) with a reference group of high parental self-efficacy (scores of ≥4). This measure has demonstrated good internal consistency (H = 0.84–0.89), and good concurrent and divergent validity [12]. | 1 year postpartum |

*Supplementary Table 2a: Risk ratios with 95% confidence intervals for any personality disorder on antenatal and one-year postpartum outcomes from available case analyses*

|  |  |  | **95% confidence interval** | |
| --- | --- | --- | --- | --- |
| **Outcome** | **n** | **RR** | **Lower** | **Upper** |
| Stressful life events (antenatal) | 327 | 1.55 | 1.11 | 2.16 |
| Social Support (antenatal) | 330 | 3.15 | 1.43 | 6.95 |
| Social Support (postpartum) | 442 | 1.51 | 0.97 | 2.37 |
| Partner relationship quality (antenatal) | 324 | 1.94 | 0.96 | 3.93 |
| Depressive symptoms (antenatal) | 331 | 2.13 | 1.09 | 4.17 |
| Depressive symptoms (postpartum) | 442 | 1.65 | 0.81 | 3.37 |
| Thoughts of self-harm (antenatal) | 331 | 0.54 | 0.07 | 4.31 |
| Thoughts of self-harm (postpartum) | 442 | 0.40 | 0.05 | 3.08 |
| Anxiety symptoms (antenatal) | 331 | 2.69 | 1.52 | 4.76 |
| Parent-infant bonding (postpartum) | 442 | 1.31 | 0.87 | 1.97 |
| Parental self-efficacy (postpartum) | 440 | 0.87 | 0.53 | 1.43 |

*Supplementary Table 2b: Risk ratios with 95% confidence intervals for total personality disorder traits on antenatal and one-year postpartum outcomes from available case analyses*

|  |  |  | **95% confidence interval** | |
| --- | --- | --- | --- | --- |
| **Outcome** | **n** | **RR** | **Lower** | **Upper** |
| Stressful life events (antenatal) | 329 | 1.07 | 0.95 | 1.21 |
| Social Support (antenatal) | 330 | 1.48 | 1.25 | 1.74 |
| Social Support (postpartum) | 442 | 1.24 | 1.12 | 1.38 |
| Partner relationship quality (antenatal) | 324 | 1.24 | 0.99 | 1.55 |
| Depressive symptoms (antenatal) | 331 | 1.34 | 1.15 | 1.57 |
| Depressive symptoms (postpartum) | 442 | 1.31 | 1.07 | 1.60 |
| Thoughts of self-harm (antenatal) | 331 | 0.85 | 0.46 | 1.58 |
| Thoughts of self-harm (postpartum) | 442 | 1.05 | 0.76 | 1.45 |
| Anxiety symptoms (antenatal) | 331 | 1.23 | 1.01 | 1.50 |
| Parent-infant bonding (postpartum) | 442 | 1.04 | 0.89 | 1.20 |
| Parental self-efficacy (postpartum) | 440 | 1.01 | 0.85 | 1.20 |

*Supplementary Table 3a: Risk ratios with 95% confidence intervals for any Cluster A personality disorder on antenatal and one-year postpartum outcomes from available case analyses*

|  |  |  | **95% confidence interval** | |
| --- | --- | --- | --- | --- |
| **Outcome** | **n** | **RR** | **Lower** | **Upper** |
| Stressful life events (antenatal) | 329 | 1.64 | 1.15 | 2.34 |
| Social Support (antenatal) | 330 | 4.18 | 1.85 | 9.42 |
| Social Support (postpartum) | 442 | 1.92 | 1.17 | 3.17 |
| Partner relationship quality (antenatal) | 324 | 2.31 | 1.02 | 5.20 |
| Depressive symptoms (antenatal) | 331 | 2.56 | 1.22 | 5.35 |
| Depressive symptoms (postpartum) | 442 | 1.64 | 0.67 | 4.01 |
| Thoughts of self-harm (antenatal) | 331 | 0.99 | 0.13 | 7.70 |
| Thoughts of self-harm (postpartum) | 442 | 1.00 | -^a^ | - ^a^ |
| Anxiety symptoms (antenatal) | 331 | 2.42 | 1.25 | 4.71 |
| Parent-infant bonding (postpartum) | 442 | 1.33 | 0.81 | 2.18 |
| Parental self-efficacy (postpartum) | 440 | 1.15 | 0.66 | 1.99 |

^a^Unable to be estimated due to small cell sizes

*Supplementary Table 3b: Risk ratios with 95% confidence intervals for any Cluster B personality disorder on antenatal and one-year postpartum outcomes from available case analyses*

|  |  |  | **95% confidence interval** | |
| --- | --- | --- | --- | --- |
| **Outcome** | **n** | **RR** | **Lower** | **Upper** |
| Stressful life events (antenatal) | 329 | 1.56 | 0.99 | 2.47 |
| Social Support (antenatal) | 330 | 4.39 | 1.85 | 10.45 |
| Social Support (postpartum) | 442 | 1.45 | 0.73 | 2.90 |
| Partner relationship quality (antenatal) | 324 | 1.90 | 0.63 | 5.67 |
| Depressive symptoms (antenatal) | 331 | 2.66 | 1.17 | 6.04 |
| Depressive symptoms (postpartum) | 442 | 2.40 | 0.92 | 6.24 |
| Thoughts of self-harm (antenatal) | 331 | 1.00 | -^a^ | -^a^ |
| Thoughts of self-harm (postpartum) | 442 | 1.00 | -^a^ | -^a^ |
| Anxiety symptoms (antenatal) | 331 | 2.81 | 1.35 | 5.86 |
| Parent-infant bonding (postpartum) | 442 | 1.18 | 0.62 | 2.23 |
| Parental self-efficacy (postpartum) | 440 | 1.18 | 0.64 | 2.17 |

^a^Unable to be estimated due to small cell sizes

*Supplementary Table 3c: Risk ratios with 95% confidence intervals for any Cluster C personality disorder on antenatal and one-year postpartum outcomes from available case analyses*

|  |  |  | **95% confidence interval** | |
| --- | --- | --- | --- | --- |
| **Outcome** | **n** | **RR** | **Lower** | **Upper** |
| Stressful life events (antenatal) | 329 | 1.24 | 0.80 | 1.93 |
| Social Support (antenatal) | 330 | 2.25 | 0.90 | 5.64 |
| Social Support (postpartum) | 442 | 1.42 | 0.82 | 2.47 |
| Partner relationship quality (antenatal) | 324 | 1.16 | 0.44 | 3.07 |
| Depressive symptoms (antenatal) | 331 | 1.15 | 0.43 | 3.06 |
| Depressive symptoms (postpartum) | 442 | 1.66 | 0.75 | 3.67 |
| Thoughts of self-harm (antenatal) | 331 | 1.00 | -^a^ | -^a^ |
| Thoughts of self-harm (postpartum) | 442 | 0.78 | 0.10 | 5.83 |
| Anxiety symptoms (antenatal) | 331 | 1.49 | 0.68 | 3.28 |
| Parent-infant bonding (postpartum) | 442 | 1.42 | 0.87 | 2.31 |
| Parental self-efficacy (postpartum) | 440 | 0.69 | 0.36 | 1.35 |

^a^Unable to be estimated due to small cell sizes

*Supplementary Table 4a: Risk ratios with 95% confidence intervals for personality difficulty compared to no personality disturbance on antenatal and one-year postpartum outcomes from available case analyses*

|  |  |  | **95% confidence interval** | |
| --- | --- | --- | --- | --- |
| **Outcome** | **n** | **RR** | **Lower** | **Upper** |
| Stressful life events (antenatal) | 329 | 0.71 | 0.44 | 1.15 |
| Social Support (antenatal) | 330 | 1.03 | 0.33 | 3.21 |
| Social Support (postpartum) | 442 | 1.23 | 0.74 | 2.05 |
| Partner relationship quality (antenatal) | 324 | 0.71 | 0.28 | 1.82 |
| Depressive symptoms (antenatal) | 331 | 0.69 | 0.27 | 1.76 |
| Depressive symptoms (postpartum) | 442 | 1.68 | 0.77 | 3.65 |
| Thoughts of self-harm (antenatal) | 331 | 1.00 | -^a^ | -^a^ |
| Thoughts of self-harm (postpartum) | 442 | 2.17 | 0.60 | 7.84 |
| Anxiety symptoms (antenatal) | 331 | 0.80 | 0.34 | 1.91 |
| Parent-infant bonding (postpartum) | 442 | 1.02 | 0.65 | 1.60 |
| Parental self-efficacy (postpartum) | 440 | 0.85 | 0.53 | 1.36 |

^a^Unable to be estimated due to small cell sizes

*Supplementary Table 4b: Risk ratios with 95% confidence intervals for simple personality disorder compared to no personality disturbance on antenatal and one-year postpartum outcomes from available case analyses*

|  |  |  | **95% confidence interval** | |
| --- | --- | --- | --- | --- |
| **Outcome** | **n** | **RR** | **Lower** | **Upper** |
| Stressful life events (antenatal) | 329 | 1.43 | 0.92 | 2.23 |
| Social Support (antenatal) | 330 | 1.78 | 0.51 | 6.15 |
| Social Support (postpartum) | 442 | 1.53 | 0.87 | 2.68 |
| Partner relationship quality (antenatal) | 324 | 1.96 | 0.86 | 4.46 |
| Depressive symptoms (antenatal) | 331 | 1.79 | 0.73 | 4.40 |
| Depressive symptoms (postpartum) | 442 | 1.37 | 0.49 | 3.82 |
| Thoughts of self-harm (antenatal) | 331 | 0.74 | 0.10 | 5.70 |
| Thoughts of self-harm (postpartum) | 442 | 0.90 | 0.11 | 7.27 |
| Anxiety symptoms (antenatal) | 331 | 2.65 | 1.30 | 5.40 |
| Parent-infant bonding (postpartum) | 442 | 1.12 | 0.63 | 2.00 |
| Parental self-efficacy (postpartum) | 440 | 0.79 | 0.39 | 1.60 |

*Supplementary Table 4c: Risk ratios with 95% confidence intervals for complex personality disorder compared to no personality disturbance on antenatal and one-year postpartum outcomes from available case analyses*

|  |  |  | **95% confidence interval** | |
| --- | --- | --- | --- | --- |
| **Outcome** | **n** | **RR** | **Lower** | **Upper** |
| Stressful life events (antenatal) | 329 | 1.45 | 0.95 | 2.22 |
| Social Support (antenatal) | 330 | 4.86 | 1.93 | 12.21 |
| Social Support (postpartum) | 442 | 1.73 | 0.89 | 3.36 |
| Partner relationship quality (antenatal) | 324 | 1.59 | 0.51 | 4.97 |
| Depressive symptoms (antenatal) | 331 | 2.15 | 0.89 | 5.21 |
| Depressive symptoms (postpartum) | 442 | 2.79 | 1.13 | 6.93 |
| Thoughts of self-harm (antenatal) | 331 | 1.00 | -^a^ | -^a^ |
| Thoughts of self-harm (postpartum) | 442 | 1.00 | -^a^ | -^a^ |
| Anxiety symptoms (antenatal) | 331 | 2.43 | 1.10 | 5.39 |
| Parent-infant bonding (postpartum) | 442 | 1.58 | 0.93 | 2.69 |
| Parental self-efficacy (postpartum) | 440 | 0.90 | 0.48 | 1.70 |

^a^Unable to be estimated due to small cell sizes

*Supplementary Table 5a: Standardised betas with 95% confidence intervals for any personality disorder on antenatal and one-year postpartum outcomes from available case analyses*

|  |  |  | **95% confidence interval** | |
| --- | --- | --- | --- | --- |
| **Outcome** | **n** | **Std. β** | **Lower** | **Upper** |
| Stressful life events (antenatal) | 327 | 0.38 | 0.00 | 0.76 |
| Social Support (antenatal) | 330 | -0.50 | -0.97 | -0.03 |
| Social Support (postpartum) | 442 | -0.27 | -0.62 | 0.08 |
| Partner relationship quality (antenatal) | 324 | -0.49 | -1.03 | 0.05 |
| Depressive symptoms (antenatal) | 331 | 0.26 | -0.13 | 0.66 |
| Depressive symptoms (postpartum) | 442 | 0.22 | -0.11 | 0.55 |
| Thoughts of self-harm (antenatal) | 331 | -0.09 | -0.33 | 0.14 |
| Thoughts of self-harm (postpartum) | 442 | -0.13 | -0.29 | 0.02 |
| Anxiety symptoms (antenatal) | 331 | 0.46 | 0.06 | 0.86 |
| Parent-infant bonding (postpartum) | 442 | 0.00 | -0.33 | 0.33 |
| Parental self-efficacy (postpartum) | 440 | 0.08 | -0.18 | 0.35 |

*Supplementary Table 5b: Standardised betas with 95% confidence intervals for total personality disorder traits on antenatal and one-year postpartum outcomes from available case analyses*

|  |  |  | **95% confidence interval** | |
| --- | --- | --- | --- | --- |
| **Outcome** | **n** | **Std. β** | **Lower** | **Upper** |
| Stressful life events (antenatal) | 327 | 0.06 | -0.07 | 0.19 |
| Social Support (antenatal) | 330 | -0.15 | -0.29 | -0.02 |
| Social Support (postpartum) | 442 | -0.12 | -0.22 | -0.01 |
| Partner relationship quality (antenatal) | 324 | -0.16 | -0.32 | 0.00 |
| Depressive symptoms (antenatal) | 331 | 0.16 | 0.02 | 0.30 |
| Depressive symptoms (postpartum) | 442 | 0.12 | -0.01 | 0.25 |
| Thoughts of self-harm (antenatal) | 331 | -0.02 | -0.09 | 0.06 |
| Thoughts of self-harm (postpartum) | 442 | 0.01 | -0.05 | 0.07 |
| Anxiety symptoms (antenatal) | 331 | 0.13 | 0.00 | 0.26 |
| Parent-infant bonding (postpartum) | 442 | 0.03 | -0.07 | 0.13 |
| Parental self-efficacy (postpartum) | 440 | 0.00 | -0.10 | 0.11 |

*Supplementary Table 6a: Standardised betas with 95% confidence intervals for any Cluster A personality disorder on antenatal and one-year postpartum outcomes from available case analyses*

|  |  |  | **95% confidence interval** | |
| --- | --- | --- | --- | --- |
| **Outcome** | **n** | **Std. β** | **Lower** | **Upper** |
| Stressful life events (antenatal) | 327 | 0.37 | -0.09 | 0.83 |
| Social Support (antenatal) | 330 | -0.76 | -1.48 | -0.03 |
| Social Support (postpartum) | 442 | -0.46 | -0.94 | 0.03 |
| Partner relationship quality (antenatal) | 324 | -0.69 | -1.50 | 0.11 |
| Depressive symptoms (antenatal) | 331 | 0.54 | -0.01 | 1.09 |
| Depressive symptoms (postpartum) | 442 | 0.26 | -0.16 | 0.68 |
| Thoughts of self-harm (antenatal) | 331 | -0.01 | -0.35 | 0.32 |
| Thoughts of self-harm (postpartum) | 442 | -0.18 | -0.29 | -0.06 |
| Anxiety symptoms (antenatal) | 331 | 0.48 | -0.02 | 0.98 |
| Parent-infant bonding (postpartum) | 442 | -0.04 | -0.50 | 0.43 |
| Parental self-efficacy (postpartum) | 440 | -0.03 | -0.38 | 0.32 |

*Supplementary Table 6a: Standardised betas with 95% confidence intervals for any Cluster B personality disorder on antenatal and one-year postpartum outcomes from available case analyses*

|  |  |  | **95% confidence interval** | |
| --- | --- | --- | --- | --- |
| **Outcome** | **n** | **Std. β** | **Lower** | **Upper** |
| Stressful life events (antenatal) | 327 | 0.74 | -0.11 | 1.60 |
| Social Support (antenatal) | 330 | -0.76 | -1.57 | 0.06 |
| Social Support (postpartum) | 442 | -0.28 | -0.87 | 0.31 |
| Partner relationship quality (antenatal) | 324 | -0.59 | -1.57 | 0.39 |
| Depressive symptoms (antenatal) | 331 | 0.47 | -0.16 | 1.10 |
| Depressive symptoms (postpartum) | 442 | 0.26 | -0.33 | 0.86 |
| Thoughts of self-harm (antenatal) | 331 | -0.18 | -0.32 | -0.05 |
| Thoughts of self-harm (postpartum) | 442 | -0.17 | -0.28 | -0.06 |
| Anxiety symptoms (antenatal) | 331 | 0.64 | -0.13 | 1.41 |
| Parent-infant bonding (postpartum) | 442 | 0.21 | -0.23 | 0.65 |
| Parental self-efficacy (postpartum) | 440 | 0.13 | -0.36 | 0.62 |

*Supplementary Table 6c: Standardised betas with 95% confidence intervals for any Cluster C personality disorder on antenatal and one-year postpartum outcomes from available case analyses*

|  |  |  | **95% confidence interval** | |
| --- | --- | --- | --- | --- |
| **Outcome** | **n** | **Std. β** | **Lower** | **Upper** |
| Stressful life events (antenatal) | 327 | 0.25 | -0.18 | 0.69 |
| Social Support (antenatal) | 330 | -0.18 | -0.60 | 0.23 |
| Social Support (postpartum) | 442 | -0.18 | -0.57 | 0.21 |
| Partner relationship quality (antenatal) | 324 | -0.10 | -0.61 | 0.42 |
| Depressive symptoms (antenatal) | 331 | 0.02 | -0.44 | 0.48 |
| Depressive symptoms (postpartum) | 442 | 0.34 | -0.11 | 0.79 |
| Thoughts of self-harm (antenatal) | 331 | -0.19 | -0.33 | -0.06 |
| Thoughts of self-harm (postpartum) | 442 | -0.08 | -0.28 | 0.12 |
| Anxiety symptoms (antenatal) | 331 | 0.10 | -0.26 | 0.47 |
| Parent-infant bonding (postpartum) | 442 | -0.08 | -0.43 | 0.27 |
| Parental self-efficacy (postpartum) | 440 | -0.03 | -0.29 | 0.23 |

*Supplementary Table 7a: Standardised betas with 95% confidence intervals for personality difficulty compared to no personality disturbance on antenatal and one-year postpartum outcomes from available case analyses*

|  |  |  | **95% confidence interval** | |
| --- | --- | --- | --- | --- |
| **Outcome** | **n** | **Std. β** | **Lower** | **Upper** |
| Stressful life events (antenatal) | 327 | -0.22 | -0.47 | 0.03 |
| Social Support (antenatal) | 330 | 0.04 | -0.20 | 0.28 |
| Social Support (postpartum) | 442 | -0.14 | -0.40 | 0.12 |
| Partner relationship quality (antenatal) | 324 | 0.03 | -0.21 | 0.27 |
| Depressive symptoms (antenatal) | 331 | -0.18 | -0.45 | 0.08 |
| Depressive symptoms (postpartum) | 442 | 0.04 | -0.25 | 0.33 |
| Thoughts of self-harm (antenatal) | 331 | -0.26 | -0.45 | -0.06 |
| Thoughts of self-harm (postpartum) | 442 | 0.14 | -0.20 | 0.47 |
| Anxiety symptoms (antenatal) | 331 | -0.08 | -0.35 | 0.19 |
| Parent-infant bonding (postpartum) | 442 | 0.08 | -0.22 | 0.38 |
| Parental self-efficacy (postpartum) | 440 | 0.08 | -0.19 | 0.35 |

*Supplementary Table 7b: Standardised betas with 95% confidence intervals for simple personality disorder compared to no personality disturbance on antenatal and one-year postpartum outcomes from available case analyses*

|  |  |  | **95% confidence interval** | |
| --- | --- | --- | --- | --- |
| **Outcome** | **n** | **Std. β** | **Lower** | **Upper** |
| Stressful life events (antenatal) | 327 | 0.13 | -0.24 | 0.51 |
| Social Support (antenatal) | 330 | -0.38 | -0.99 | 0.22 |
| Social Support (postpartum) | 442 | -0.26 | -0.71 | 0.19 |
| Partner relationship quality (antenatal) | 324 | -0.56 | -1.25 | 0.14 |
| Depressive symptoms (antenatal) | 331 | 0.04 | -0.46 | 0.54 |
| Depressive symptoms (postpartum) | 442 | 0.10 | -0.29 | 0.49 |
| Thoughts of self-harm (antenatal) | 331 | -0.08 | -0.46 | 0.30 |
| Thoughts of self-harm (postpartum) | 442 | -0.06 | -0.27 | 0.14 |
| Anxiety symptoms (antenatal) | 331 | 0.46 | -0.10 | 1.02 |
| Parent-infant bonding (postpartum) | 442 | 0.04 | -0.43 | 0.51 |
| Parental self-efficacy (postpartum) | 440 | 0.15 | -0.20 | 0.50 |

*Supplementary Table 7c: Standardised betas with 95% confidence intervals for complex personality disorder compared to no personality disturbance on antenatal and one-year postpartum outcomes from available case analyses*

|  |  |  | **95% confidence interval** | |
| --- | --- | --- | --- | --- |
| **Outcome** | **n** | **Std. β** | **Lower** | **Upper** |
| Stressful life events (antenatal) | 327 | 0.58 | -0.11 | 1.26 |
| Social Support (antenatal) | 330 | -0.61 | -1.34 | 0.11 |
| Social Support (postpartum) | 442 | -0.37 | -0.89 | 0.15 |
| Partner relationship quality (antenatal) | 324 | -0.39 | -1.23 | 0.46 |
| Depressive symptoms (antenatal) | 331 | 0.43 | -0.17 | 1.03 |
| Depressive symptoms (postpartum) | 442 | 0.42 | -0.11 | 0.94 |
| Thoughts of self-harm (antenatal) | 331 | -0.26 | -0.45 | -0.06 |
| Thoughts of self-harm (postpartum) | 442 | -0.15 | -0.27 | -0.02 |
| Anxiety symptoms (antenatal) | 331 | 0.41 | -0.15 | 0.97 |
| Parent-infant bonding (postpartum) | 442 | 0.00 | -0.41 | 0.41 |
| Parental self-efficacy (postpartum) | 440 | 0.04 | -0.34 | 0.42 |

**References**

1. Brugha TS, Cragg D (1990) The List of Threatening Experiences: the reliability and validity of a brief life events questionnaire. Acta Psychiatr Scand 82 (1):77-81. doi:10.1111/j.1600-0447.1990.tb01360.x

2. Webster J, Linnane JW, Dibley LM, Hinson JK, Starrenburg SE, Roberts JA (2000) Measuring social support in pregnancy: can it be simple and meaningful? Birth 27 (2):97-101

3. Webster J, Pritchard MA, Creedy D, East C (2003) A simplified predictive index for the detection of women at risk for postnatal depression. Birth 30 (2):101-108. doi:10.1046/j.1523-536x.2003.00228.x

4. Spanier GB (1976) Measuring dyadic adjustment: New scales for assessing the quality of marriage and similar dyads. Journal of Marriage and the Family:15-28

5. Cox JL, Holden JM, Sagovsky R (1987) Detection of Postnatal Depression: Development of the 10-item Edinburgh Postnatal Depression Scale. British Journal of Psychiatry 150 (6):782-786. doi:10.1192/bjp.150.6.782

6. Murray D, Cox JL (1990) Screening for depression during pregnancy with the edinburgh depression scale (EDDS). Journal of Reproductive and Infant Psychology 8 (2):99-107. doi:10.1080/02646839008403615

7. Lewis G, Pelosi AJ, Araya R, Dunn G (1992) Measuring psychiatric disorder in the community: a standardized assessment for use by lay interviewers. Psychol Med 22 (2):465-486. doi:10.1017/s0033291700030415

8. Patton GC, Coffey C, Posterino M, Carlin JB, Wolfe R, Bowes G (1999) A computerised screening instrument for adolescent depression: population-based validation and application to a two-phase case-control study. Social Psychiatry and Psychiatric Epidemiology 34 (3):166-172. doi:10.1007/s001270050129

9. Brockington IF, Fraser C, Wilson D (2006) The Postpartum Bonding Questionnaire: a validation. Arch Womens Ment Health 9 (5):233-242. doi:10.1007/s00737-006-0132-1

10. Mathews TL, Emerson MR, Moore TA, Fial A, Hanna KM (2019) Systematic Review: Feasibility, Reliability, and Validity of Maternal/Caregiver Attachment and Bonding Screening Tools for Clinical Use. Journal of Pediatric Health Care 33 (6):663-674. doi:10.1016/j.pedhc.2019.04.018

11. Zubrick SR, Lucas, N., Westrupp, E. M. & Nicholson, J. M (2014) Parenting measures in the Longitudinal Study of Australian Children: Construct validity and measurement quality, Waves 1 to 4. . LSAC technical paper 12:1-110

12. Zubrick SR, Lucas N, Westrupp EM, Nicholson JM (2014) Parenting measures in the Longitudinal Study of Australian Children: Construct validity and measurement quality, Waves 1 to 4. Canberra: Department of Social Services 14697610 (2024):12
